# Effects of preoperative statin use on perioperative outcomes of carotid endarterectomy

**DOI:** 10.1002/brb3.597

**Published:** 2016-11-05

**Authors:** Enzo Ballotta, Antonio Toniato, Filippo Farina, Claudio Baracchini

**Affiliations:** ^1^ The Vascular Surgery GroupDepartment of SurgicalOncological and Gastroenterological SciencesSchool of MedicineUniversity of PaduaPadovaItaly; ^2^ Stroke Unit and Neurosonology LaboratoryDepartment of NeurosciencesSchool of MedicineUniversity of PaduaPadovaItaly

**Keywords:** carotid endarterectomy, carotid restenosis, eversion technique, statin therapy, stroke

## Abstract

**Objectives:**

Several studies have shown the beneficial role of statins in reducing the risk of major perioperative complications and death associated with noncardiac vascular surgery, but few have focused on their effects in the event of carotid endarterectomy (CEA). This study analyzes the effects of preoperative statin use on perioperative outcomes in patients undergoing CEA.

**Materials and Methods:**

Data from all consecutive patients who underwent primary CEA for symptomatic and asymptomatic carotid disease between 2002 and 2014 at a single institution were prospectively stored in a vascular surgery registry, recording risk factors, medication, and indication for surgery. Endpoints of the study were perioperative (30‐day) stroke and death.

**Results:**

Overall, 784 patients were on statins (825 CEAs, Group I), while 494 were not (545 CEAs, Group II). There were two perioperative strokes in Group I (0.24%) and four in Group II (0.73%; *p* = .22), and no deaths. The only nonfatal cardiac complication occurred in Group II (0.18%, *p* = .39). A neurologist assessed patients at 1, 6, and 12 months after CEA, and every 2 years thereafter. Follow‐up (range: 0.1–13 years; mean, 6.3 ± 3.7 years) was obtained for 1,239 patients (1,326 CEAs). Because 165 patients (166 CEAs) crossed over from Group II to Group I during the follow‐up time, long‐term data were stratified by postoperative statin treatment rather than by preoperative statin use. The 5‐ and 10‐year restenosis/occlusion and survival rates did not differ significantly between the two groups.

**Conclusions:**

Taking statins prior to CEA did not seem to affect the risk of major perioperative ischemic events and death, most likely due to the extremely low overall incidence of perioperative complications.

## 1 Introduction

Carotid endarterectomy (CEA) remains the “gold standard” treatment for severe symptomatic and asymptomatic carotid disease with a view to preventing cerebrovascular ischemic events (Chambers, You, & Donnan, 2005; Rothwell et al., 2003). On the other hand, since the first large randomized controlled trials (RCTs) were conducted (Executive Committee for the Asymptomatic Carotid Atherosclerosis Study, 1995; North American Symptomatic Carotid Endarterectomy Trial Collaborators, 1991), significant improvement has been made in the medical management of patients with atherosclerotic carotid disease and there is now compelling evidence of a drop in the risk of cerebral ischemic events (Spence, 2010). In efforts to identify the best medical therapy, 3‐hydroxy‐3‐methyl‐glutaryl coenzyme A reductase inhibitors (statins) have been used effectively, alongside latest generation antiplatelet and antihypertensive drugs, better glycemic control, and lifestyle changes. The exact mechanisms behind the beneficial effects are uncertain and highly speculative, but a growing body of data suggests that, next to the cholesterol and low‐density lipoprotein reduction, statin treatment has numerous pleiotropic effects (Halcox & Deanfield, 2004). It would modulate various inflammatory responses involved in the initiation and progression of atherosclerotic disease, and it would also have other cellular effects, such as reducing platelet adhesion to improve fibrinolysis, reducing thrombosis, improving endothelial cell function, upregulating endothelial nitric oxide synthase, stabilizing plaque, reducing vascular smooth muscle cell proliferation and migration, and also have a neuroprotective role (Abela et al., 2011; Kong & Zhu, 2012; O'Neil‐Callahan et al., 2005; Paraskevas et al., 2007; Sadowitz, Meier, & Gahtan, 2010; Yla‐Herttuala et al., 2013). Many clinical studies, including several RCTs, have clearly demonstrated that statins were effective on primary and secondary protection against adverse cardiovascular events in many patients, even those with normal lipid levels, in cases of cerebral, peripheral, and coronary arterial disease, in reducing the incidence of stroke and myocardial infarction, thus improving overall survival (Amarenco & Labreuche, 2009; Downs et al., 1998; Heart Protection Study Collaborative Group, 2004; O'Regan, Wu, Arora, Perri, & Mills, 2008; Scandinavian Simvastatin Survival Study Group, 1994; Shepherd et al., 1995; Sillesen et al., 2008; The Long‐Term Intervention with Pravastatin in Ischemic Disease (LIPID) Study Group, 1998). Moreover, recent reports have shown that the preoperative use of statins has a protective role after noncardiac vascular and cardiac surgery, attenuating the perioperative incidence of stroke, myocardial infarction (MI), and death (Antoniou et al., 2015; Chopra et al., 2012; Kapoor, Kanji, Buckingham, Devereaux, & McAlister, 2006; Winchester, Wen, Xie, & Bavry, 2010). Only few studies, however, have investigated on the benefits of statin use in patients undergoing CEA, reporting variable results (AbuRahma et al., 2015; Brooke et al., 2007; Kennedy, Quan, Buchan, Ghali, & Feasby, 2005; LaMuraglia et al., 2005; McGirt et al., 2005; Perler, 2007a, 2007b). This observational study was undertaken to analyze the effects of statin therapy on perioperative outcomes after CEA.

## 2 Methods

Our Institutional Review Board and Ethics Committee approved the study. All patients gave their written informed consent to the analysis of their records and the publication of the findings.

Details of all consecutive patients undergoing primary CEA at our tertiary referral center between 2002 and 2014 for symptomatic and asymptomatic carotid stenosis—according to the North American Symptomatic Carotid Endarterectomy Trial Collaborators (1991) and the Asymptomatic Carotid Atherosclerotic Study (Executive Committee for the Asymptomatic Carotid Atherosclerosis Study, 1995) criteria, respectively—were prospectively stored in a vascular surgery registry. Symptomatic disease was documented by a vascular neurologist when patients experienced either an ischemic cerebral event, i.e., a *transient ischemic attack* (TIA), defined as temporary hemispheric symptoms lasting no more than 24 hr, with complete recovery; a *stroke*, categorized as *minor* (no disabling) *stroke*, defined by a score <3 on the modified Rankin‐scale and *major* (disabling) *stroke*, defined by a score >3; or an ocular ischemic event, defined as a transient monocular visual loss (*amaurosis fugax*). Patients scheduled for CEA with concomitant coronary artery bypass grafting or concurrent surgery for associated supra‐aortic trunk lesions, and patients who underwent redo‐CEA were excluded from the present analysis. All patients' demographic and clinical data were recorded on a standardized form, including potential atherosclerotic risk factors, anatomical and clinical variables, preoperative medication, details of surgery, and all perioperative outcomes. All patients with an ultrasound diagnosis of a hemodynamically significant carotid lesion underwent a confirmatory noninvasive neuroradiological imaging by magnetic resonance (MR) angiography (MRA) or computed tomography (CT) angiography (CTA). Arterial digital subtraction angiography was performed only in case of carotid pseudo‐occlusion or disagreement between ultrasonographic study and MRA/CTA. All tests were performed using a high‐resolution, color‐coded duplex sonography scanner (the Acuson Sequoia 512 ultrasound system up until 2008, and the Philips iU 22 from 2008 onward) with a high‐frequency (5–10 MHz) linear probe for assessing cervical vessels, and a low‐frequency (2–4 MHz) sectorial probe for assessing intracranial arteries. Stenoses were graded according to velocity criteria reported elsewhere, and validated in our accredited vascular laboratory (Ballotta et al., 2008). Patients taking 3‐hydroxy‐3‐methyl‐glutaryl coenzyme A reductase inhibitors at any dosage for at least 2 weeks before surgery were classified as statin users (Group I). Statins were all assumed to have the same effect on the outcome, and were thus counted together as a single variable. All patients who were not on statins at the time of their preoperative evaluation formed our control group (Group II).

Preoperative patient preparation was standardized. The preoperative cardiac work‐up was tailored to each individual's clinical history, electrocardiographic (ECG) findings, and symptoms. Patients with evidence of clinically important coronary artery disease underwent echocardiography or dipyridamole‐thallium stress tests followed by coronary arteriography, as indicated.

All surgical procedures were eversion CEAs performed by the same surgeon in patients under general anesthesia, with routine intraoperative electroencephalographic monitoring for a selective use of intraluminal shunting (Baracchini et al., 2012). All our patients were monitored in the recovery room for 2 hr until their blood pressure and neurological status were judged acceptable; they were then transferred to a nursing unit specialized in vascular care where their vital parameters (including cardiac) were monitored for the next 12–24 hr after surgery.

All patients on statins before surgery were discharged on statin therapy. Every effort was made to discharge patients of the control group on statins, sending a note to the referring physicians to advise them on the importance of statin therapy. Because many patients crossed over from Group II to Group I during the follow‐up time, long‐term data were stratified by postoperative statin treatment rather than by preoperative statin use.

### 2.1 Surveillance protocol

Immediately after CEA, patients' vital signs and neurological status were recorded. All patients were scheduled for regular clinical check‐ups after 1, 6, and 12 months, and then every 2 years. At each visit, patients systematically had a physical examination and a neurological assessment by a neurologist, and concomitant duplex ultrasound scans performed by two experienced neurosonographers. Restenoses ≥50% and ≥70% were analyzed in both groups of patients. A peak systolic velocity (PSV) of more than 130 cm/s with spectral broadening throughout the systole, and an increased peak diastolic velocity were consistent with a stenosis ≥50% diameter reduction, while a PSV greater than 240 cm/s was consistent with ≥70% stenosis. Any stenosis ≥70% identified on duplex ultrasound scanning was confirmed by CT/MR angiography. The ultrasound follow‐up schedule was modified if any progressing or severe lesions were detected, or if patients became symptomatic. New neurological events after CEA were always classified by the neurologist and confirmed by noninvasive brain imaging. All patients who suffered a perioperative ischemic event underwent brain MR imaging in order to confirm the clinical diagnosis, gather information on stroke mechanism, and document the extension of the cerebral infarct. Cardiac complications were classified by a single cardiologist and included: (i) MI with a diagnosis based on creatinine kinase–MB levels and ECG findings, (ii) pulmonary edema confirmed by chest X‐ray, (iii) documented ventricular fibrillation or primary cardiac arrest, and (iv) new congestive heart failure requiring a pacemaker. A postoperative ECG was routinely obtained in all patients with a history of coronary artery disease, congestive heart failure, or arrhythmia (rhythm other than sinus) and cardiac isoenzymes were surveyed in all patients who had new findings at postoperative ECG. Any complications and events observed during the follow‐up were recorded in accordance with the guidelines of the Reporting Standards for Carotid Interventions from the Society for Vascular Surgery (Timaran, McKinsey, Schneider, & Littooy, 2011). Primary endpoints were perioperative stroke and death.

### 2.2 Statistical analysis

The statistical analysis was performed with the SSPS statistical software (SPSS version 12.0.1, SPSS, Inc., Chicago, IL, USA). Patients' demographic data are given as medians, means, and ranges, baseline clinical and diagnostic findings in terms of incidence rates. Frequencies and categorical data were compared with χ^2^ or Fisher's exact test, as appropriate, calculating the odds ratio (OR) with 95% confidence intervals (CIs). Freedom from late restenosis/occlusion and cerebral ischemic events, and survival rates were calculated using the Kaplan–Meier method and are reported as “life‐table” analyses. Significance was assumed at *p *< .05. Several perioperative data and technical details, such as death, stroke, electroencephalographic changes, shunt placement, nerve injury, and neck hematoma, were analyzed vis‐à‐vis surgical procedures rather than patients because each perioperative outcome was correlated with the CEA procedure, and patients who underwent bilateral CEAs were exposed to twice the risk of stroke, death, or other complications.

## 3 Results

Overall, 1,278 patients (784 of them on statins) underwent 1,370 CEAs (92 were staged bilateral CEAs and 41 of these were in Group I). Patient's demographic details, risk factors, and indications for CEA are summarized in Table 1. The proportion of patients between 70 and 80 years old was significantly higher in the group on statins (*p* = .002), while patients over 80 years old were less likely to be on statins (*p* = <.001). Patients on statins more often had a history of cardiac disease (*p* = .003), symptomatic carotid disease (*p* = .003), and cerebral ischemic events (*p* = .003). The mean carotid cross‐clamping time was significantly longer for patients on statins (*p* < .001), as shown in Table 2.

**Table 1:** Baseline characteristics

	Total (*n* = 1,278)	Group I (*n* = 784)	Group II (*n* = 494)	*p* value
CEA procedures	1,370 (100)	825 (60.2)	545 (39.8)	
Mean age, years (±SD)		75.7 (5.6)	76.1 (4.8)	.19
<70	406 (31.8)	251 (32.0)	155 (31.4)	.81
70–80	627 (49.1)	412 (52.5)	215 (45.5)	.002
>80	245 (19.1)	121 (15.4)	124 (25.1)	<.001
Male	878 (68.7)	549 (70.0)	329 (66.6)	.19
Risk factors				
Hypertension^a^	749 (58.6)	472 (60.2)	277 (56.1)	.14
Smoking^b^	864 (67.6)	543 (69.2)	321 (65.0)	.11
Diabetes	427 (33.4)	273 (34.8)	154 (31.2)	.18
Hyperlipidemia^c^	578 (45.2)	355 (45.2)	223 (45.1)	.96
Cardiac disease	604 (47.3)	396 (50.5)	208 (42.1)	.003
CKD	106 (8.3)	61 (7.8)	45 (9.1)	.40
Pulmonary disease	207 (16.2)	126 (16.0)	81 (16.3)	.87
Symptoms	921 (67.2)	580 (70.3)	341 (62.5)	.003
Cerebral events	748 (54.6)	477 (57.8)	271 (49.7)	.003
TIA	425 (31.0)	268 (32.5)	157 (28.8)	.15
Stroke	323 (23.6)	209 (25.3)	114 (21.0)	.06
Ocular events	173 (12.6)	103 (12.5)	70 (12.8)	.84
No symptoms	449 (32.8)	245 (29.6)	204 (37.4)	.003

CEA, carotid endarterectomy; CO, contralateral occlusion; CKD, chronic kidney disease; SD, standard deviation; TIA, transient ischemic attack.

Values within parentheses represent percentages.

a Arterial pressure >140/90 mmHg or blood pressure treated with medication.

b Current or cessation within the past 5 years.

c Serum concentration of cholesterol <6.5 mmol/L or triglycerides >2.0 mmol/L.

**Table 2:** Degree of carotid lesions, concomitant medication, and intraoperative details

Variable	Total	Group I	Group II	OR (95% CI)	*p* value
% Ipsilateral stenosis					
<50%	39 (2.9)	25 (3.0)	14 (2.6)	1.18 (0.586–2.424)	.61
50%–59%	17 (1.2)	10 (1.2)	7 (1.3)	0.94 (0.329–2.759)	.90
60%–69%	99 (7.2)	58 (7.0)	41 (7.5)	0.93 (0.602–1.438)	.73
70%–79%	590 (43.1)	357 (43.2)	233 (42.7)	1.02 (0.816–1.279)	.84
80%–89%	460 (33.6)	278 (33.7)	182 (33.4)	1.01 (0.801–1.283)	.90
90%–99%	165 (12.0)	97 (11.7)	68 (12.5)	0.93 (0.663–1.319)	.69
Contralateral disease					
<60%	912 (71.4)	568 (72.5)	344 (69.6)	1.14 (0.888–1.480)	.28
≥60%	154 (12.0)	91 (11.6)	63 (12.8)	0.89 (0.629–1.284)	.54
Occlusion	212 (16.6)	125 (15.9)	87 (17.6)	0.88 (0.650–1.212)	.44
Concomitant medication					
Antiplatelet treatment	1,178 (92.2)	722 (92.1)	456 (92.3)	0.97 (0.624–1.507)	.88
Clopidogrel	377 (29.5)	224 (28.6)	153 (30.9)	0.89 (0.692–1.149)	.36
Clopidogrel plus ASA	171 (13.4)	111 (14.1)	60 (12.1)	1.19 (0.841–1.694)	.30
ASA	414 (32.3)	265 (34.0)	149 (30.2)	1.18 (0.921–1.518)	.17
Ticlopidine	181 (14.1)	105 (13.4)	76 (15.4)	0.85 (0.611–1.185)	.32
Dipyridamole	35 (2.7)	17 (2.2)	18 (3.6)	0.58 (0.285–1.204)	.11
Anticoagulant (warfarin)	118 (9.2)	73 (9.3)	45 (9.1)	1.02 (0.682–1.541)	.90
Intraoperative variables					
Left side of operation	741 (54.1)	456 (55.3)	285 (52.3)	1.12 (0.902–1.409)	.27
Shunt placement	178 (13.0)	103 (12.5)	75 (13.8)	0.89 (0.642–1.246)	.49
Carotid clamping time, min (±SD)		18 ± 6	15 ± 5	3.00 (2.39–3.61)	<.001

ASA, acetyl salicylic acid; SD, standard deviation.

Values within parentheses represent percentages.

### 3.1 Perioperative (30‐day) results

Overall, there were 6 (0.43%) perioperative ipsilateral strokes and no deaths (Table 3). In all cases, the strokes occurred in symptomatic patients (2/580, 0.34% for those on statins vs. 4/341, 1.17% for those no‐on statins [*p* = .20]), with a proportion of 0.24% (2/825) in Group I versus 0.73% (4/545) in Group II, an absolute risk difference of 0.5% (*p* = .22).

**Table 3:** Perioperative (30‐day) results

	Total (*n* = 1,370)	Group I (*n* = 825)	Group II (*n* = 545)	OR (95% CI)	*p* value
Stroke	6 (0.43)	2 (0.24)	4 (0.73)	0.32 (0.042–2.081)	.22^a^
Major	4 (0.29)	1 (0.12)	3 (0.55)	0.21 (0.009–2.352)	.30^a^
Minor	2 (0.14)	1 (0.12)	1 (0.18)	0.66 (0.018–24.15)	.63^a^
Death	0				
Cardiac complication	1 (0.07)	0	1 (0.18)	0.00 (0.00–11.44)	.39^a^
Nerve injury	41 (2.99)	23 (2.78)	18 (3.30)	0.84 (0.431–1.642)	.58
Neck hematoma	45 (3.28)	23 (2.78)	22 (4.03)	0.68 (0.362–1.285)	.20

Values within parentheses represent percentages.

a Fisher's exact test.

### 3.2 Other complications

The only nonfatal cardiac complication occurred in Group II, with an absolute risk difference of 0.18% (*p* = .39), and it was managed successfully with medication. Other surgical morbidities included 41 (2.99%) nerve injuries, involving the cranial nerves in 32 (2.33%) cases, and the cervical nerves in 9 (0.66%), and 45 (3.3%) neck hematomas requiring surgical re‐exploration but causing no further complications. No statistically significant differences emerged between the two groups (Table 3).

### 3.3 Late outcomes

**Figure 1:**
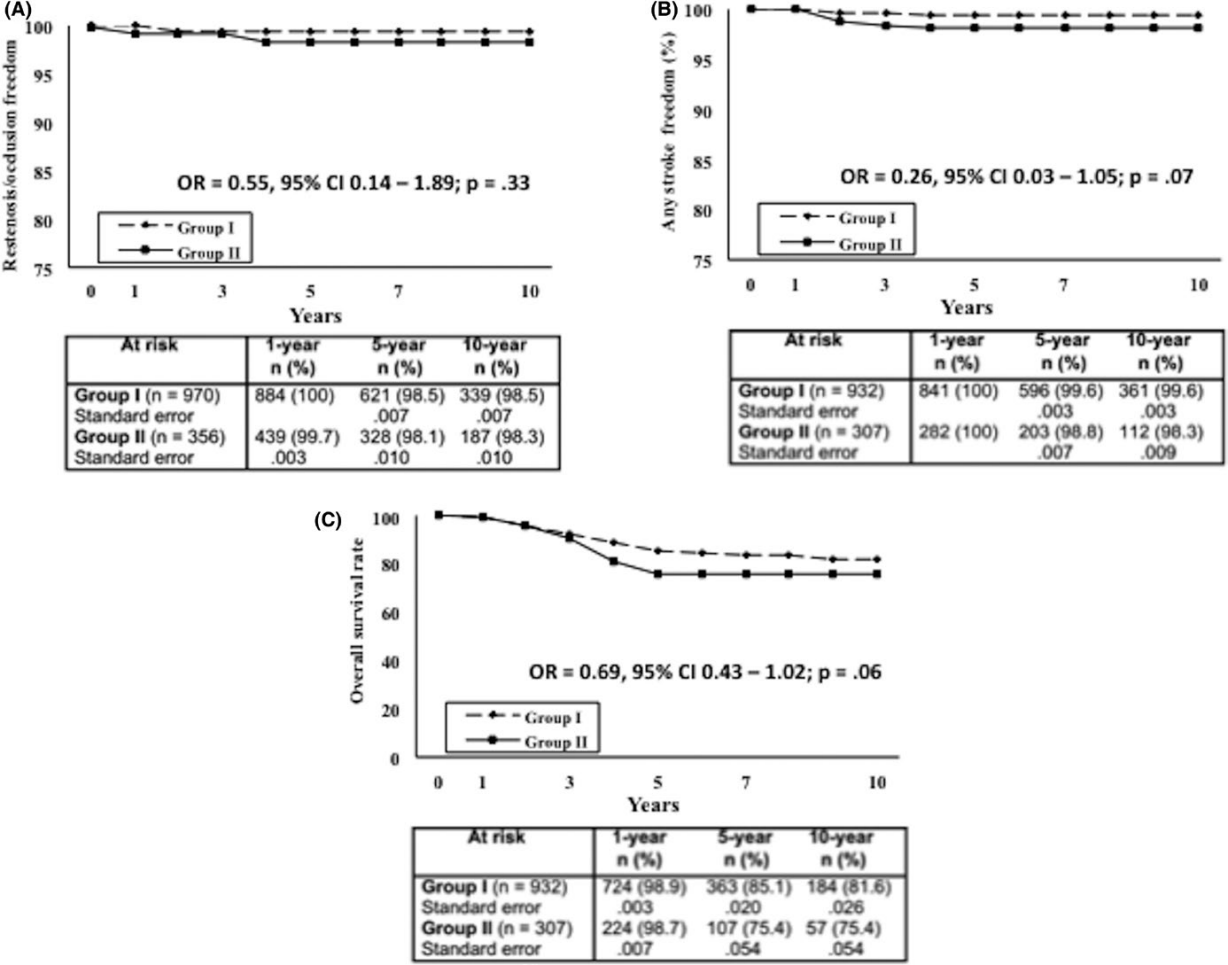
(A) Restenosis/occlusion freedom, (B) any late stroke freedom, and (C) overall survival rates of Groups I and II who underwent carotid endarterectomy. Values are presented as number (%)

Among the 1,278 patients alive 30 days after surgery, 39 (3.0%; 44 CEAs) were lost to follow‐up (17 patients in Group I, 2.1%; 21 CEAs and 22 patients in Group II, 4.4%; 23 CEAs). A complete follow‐up (range: 0.1–13 years; mean, 6.3 ± 3.7 years) was thus obtained for 1,239 patients (96.9%) and 1,326 CEAs (96.7%). Because 165 patients (166 CEAs) crossed over from Group II to Group I during the follow‐up time, data for Group I (932 patients, 970 CEAs) and Group II (307 patients, 356 CEAs) were stratified by postoperative statin treatment rather than by preoperative statin use (Table 4). Overall, only one carotid occlusion was detected (0.09%): it occurred within the first postoperative year in an asymptomatic male in Group II, and involved an unshunted vessel that had been found patent at the first two ultrasound scans (Table 4). Altogether, 10 restenoses ≥50% (0.75%) were detected (6 [0.61%] in Group I and 4 [1.12%] in Group II, *p* = .47; OR 0.54, 95% CI = 0.136–2.319) that involved unshunted vessels and occurred without any symptoms, mainly within 24 months of surgery. Two of these stenoses (0.15%) were ≥70% (both in Group II; *p* = .07): the first remained stable at subsequent ultrasound scans and was thus treated conservatively, while the other rapidly progressed, becoming severe enough to require a second CEA procedure 19 months after the first revascularization. At 1, 5, and 10 years, Kaplan–Meier analysis showed that the rates of freedom from restenosis/occlusion were 100%, 98.5 ± 0.7%, and 98.5 ± 0.7% for Group I, as opposed to 99.7 ± 0.3%, 98.1 ± 1.0%, and 98.1 ± 1.0% for Group II (OR, 0.55; 95% CI 0.14–1.89, *p* = .33; Figure 1a).

**Table 4:** Long‐term results

Outcomes	Total	Group 1	Group 2	OR (95% CI)	*p* value
Stroke	7 (0.52)	3 (0.30)	4 (1.12)	0.27 (0.048–1.445)	.09^a^
Ipsilateral	5 (0.37)	2 (0.20)	3 (0.84)	0.24 (0.028–1.787)	.12^a^
Contralateral	2 (0.15)	1 (0.10)	1 (0.28)	0.36 (0.010–13.41)	.46^a^
Death	118 (9.5)	81 (8.69)	37 (12.05)	0.69 (0.451–1.072)	.08
Stroke related	2 (0.16)	2 (0.21)	0	Inf (0.081–Inf)	.56^a^
MI related	57 (4.6)	39 (4.2)	18 (5.9)	0.70 (0.381–1.293)	.22
Carotid restenoses	10 (0.75)	6 (0.61)	4 (1.12)	0.54 (0.136–2.319)	.47^a^
50%–69%	8 (0.60)	6 (0.61)	2 (0.56)	1.10 (0.201–7.920)	.63^a^
≥70%	2 (0.15)	0	2 (0.56)	0.00 (0.00–1.487)	.07^a^
Carotid occlusion	1 (0.09)	0	1 (0.28)	0.00 (0.00–6.356)	.26^a^
All carotid restenoses/occlusions	11 (0.7)	6 (0.61)	5 (1.40)	0.43 (0.118–1.657)	.16

MI, myocardial infarction.

a Fisher's exact test.

Values within parentheses represent percentages.

No significant differences emerged between the two groups when patients were stratified within each group by the presence or absence of symptoms at presentation.

Overall, there were seven late strokes (0.52%), three of these in Group I (0.30%, *p* = .09; OR 0.27, 95% CI = 0.048–1.445) and none of them occurred in patients with recurrent stenosis. Three were cardioembolic and two were lacunar (one contralateral to the operated side), while two (ipsilateral to the operated side and contralateral to a carotid occlusion) were probably hemodynamic in nature, judging from the CT images (Table 4). At 1, 5, and 10 years, Kaplan–Meier analysis showed that the rates of freedom from stroke were 100%, 99.6 ± 0.3%, and 99.6 ± 0.3% for Group I and 100%, 98.8 ± 0.7%, and 98.38 ± 0.9% for Group II (OR 0.26; 95% CI 0.03–1.05, *p* = .07; Figure 1b). Therefore, despite a trend toward improving outcome in Group I patients, it failed to reach statistical significance.

No significant differences emerged between the groups when patients were stratified within each group by the presence or absence of symptoms at presentation.

There were 118 late deaths (9.5%; 8.7% in Group I vs. 12.0% in Group II, *p* = .08; OR 0.69, 95% CI = 0.451–1.072) in the series as a whole. The cause was primarily cardiac related (*n* = 66, 55.9%) and due to MI (*n* = 57), ventricular fibrillation (*n* = 3), and congestive heart failure (*n* = 6). No significant difference emerged between the groups when the incidence of MI was considered (39, 4.2% for Group I vs. 18, 5.9% for Group II: *p* = .22; OR 0.70, 95% CI = 0.38–1.29). Two deaths were stroke related (1.7%)—one involving a female patient with atrial fibrillation of recent onset, the other contralateral to the revascularized side and ipsilateral to a carotid occlusion (Table 4). At 1, 5, and 10 years, the survival rates were 98.9 ± 0.3%, 85.1 ± 2.0%, and 81.6 ± 2.6% for Group I and 98.7 ± 0.7%, 75.4 ± 5.4%, and 75.4 ± 5.4% for Group II (OR, 0.69; 95% CI = 0.43–1.02, *p* = .06; Figure 1c). Therefore, despite a trend toward improving outcome in Group I patients, it failed to reach statistical significance.

## 4 Discussion

Large RCTs in symptomatic and asymptomatic patients support the safety and efficacy of CEA and its superiority over the best medical management of carotid disease (Chambers et al., 2005; Executive Committee for the Asymptomatic Carotid Atherosclerosis Study, 1995; North American Symptomatic Carotid Endarterectomy Trial Collaborators, 1991; Rothwell et al., 2003). Although the incidence of CEA‐related perioperative stroke and death has dropped considerably in the past two decades, there is always a small but non‐negligible risk of perioperative cerebral ischemic events occurring even when CEA is performed at centers achieving excellent outcomes. Any pharmacological intervention aimed at reducing the incidence of perioperative complications is therefore worth investigating, since it might further increase the potential benefit of the surgical procedure.

The results of our study showed that using statins before CEA did not significantly affect the incidence of perioperative cerebral ischemic events or death, when considered as independent variables or examined in combination. The crude incidence of perioperative cerebral ischemic events was nearly three times lower in patients on statins (0.24% vs. 0.73%, *p* = .22), and the fact that this trend failed to reach statistical significance was likely due to the negligible overall perioperative stroke rate. These findings correlate well with other clinical investigations (AbuRahma et al., 2015; Sanders, Nicholson, Lewis, Smith, & Alderson, 2013), but are in conflict with the commonly held conviction that statin therapy has a beneficial influence on perioperative risks in patients undergoing CEA.

In a recent Cochrane Collaboration Systematic Review examining pooled data from three trials with a total of 178 patients, analyses failed to demonstrate any beneficial effects of the preoperative use of statins on vascular surgical procedures, including CEA. Given the limited amount of data obtained from the RCTs examined, due to the strict inclusion criteria adopted, the authors recommended that further investigations be conducted to gather better information (Sanders et al., 2013). A recently published retrospective analysis on 500 patients (299 on statins) who underwent CEA for symptomatic and asymptomatic carotid disease revealed no significant difference between patients who were or were not on statins when perioperative stroke, cardiac complication, or death rates were compared, independently or in combination (AbuRahma et al., 2015). Nor did any statistically significant difference emerge between statin users and nonusers when the incidence of late stroke and death was considered, although the overall early and late mortality was nearly 50% lower in patients on statins (2.3% vs. 4.5%, *p* = .18), with a decrease of nearly 75% in diabetic patients (2.5% vs. 8.5%, *p* = .11) and nearly 50% in those with hypercholesterolemia (2.2% vs. 4.3%, *p* = .31).

Most of the available information on the association between statin therapy prior to surgery and a significant reduction of perioperative adverse events after CEA comes from the clinical data generated by the Johns Hopkins group (which emphasized the beneficial effects of statin therapy in four different papers (Brooke et al., 2007; McGirt et al., 2005; Perler, 2007a, 2007b), and from a large series of CEAs performed at several hospitals in Western Canada (Kennedy et al., 2005). The Johns Hopkins experience concerned 1,556 patients (657 on statins) who underwent CEA for symptomatic and asymptomatic disease performed by 13 attending surgeons over a decade (McGirt et al., 2005; Perler, 2007b; Brooke et al., 2007): the perioperative stroke and death rates were significantly lower for patients on statins than for those who were not (1.2% vs. 4.5%, *p* < .01 and 0.3% vs. 2.1%, *p* < .01, respectively). The authors attributed their results mainly to the pleiotropic effects of statins in stabilizing carotid plaque that would otherwise be disrupted, embolize, and lead to a perioperative adverse event. The Canadian study involved 2,031 symptomatic patients (815 on statins) and 1,252 asymptomatic patients (655 on statins) who underwent CEA over a 2‐year period (Kennedy et al., 2005): symptomatic patients who were on statins had considerably better perioperative outcomes than those who were not, in terms of in‐hospital stroke/death (OR 0.55, 95% CI 0.32–0.95) or death (OR 0.25, 95% CI 0.07–0.90). Remarkably, no statistically significant difference in stroke and mortality rates was associated with statin use in asymptomatic patients (Kennedy et al., 2005). It may be that the beneficial effects of statins reported in such patients are actually the result of a generally more comprehensive and aggressive medical treatment, and possibly of a greater compliance with such treatment. Despite the retrospective and observational nature of both these clinical observations, some authors have suggested that statin administration should be recommended for all patients before CEA (Perler, 2007a, 2007b).

As in other investigations (AbuRahma et al., 2015), we found that statin therapy did not significantly affect the incidence of late restenosis/occlusion. This was not unexpected because the occurrence of post‐CEA restenosis relates mainly to how the arteriotomy is closed. All of our patients underwent eversion CEA, which has commonly been identified as an independent factor contributing to better long‐term results, although there is currently no evidence of the superiority of one carotid surgical technique over another (CEA with routine patching vs. eversion CEA). In another report in which all patients had traditional CEA routinely patched (AbuRahma et al., 2015), a restenosis ≥50% occurred more frequently among patients who were on statins than among those who were not, although the difference was not statistically significant (3.7% vs. 2.9%, *p* = .64). On the other hand, a retrospective study on 2,127 traditional selectively patched CEAs performed over a 10‐year period identified a 5.8% incidence of late anatomical failure: at multivariate analysis, the concurrent use of lipid‐lowering drugs, including independently statins (*p* < .002) and no statin lipid‐lowering drugs (*p* < .03), was the only factor protecting against recurrent stenosis/occlusion (LaMuraglia et al., 2005).

Interestingly, the carotid cross‐clamping time in our series was significantly longer for patients taking statins, since removing the carotid plaque in such patients is quite troublesome, due almost exclusively to a greater difficulty in finding the right cleavage plane for everting the adventitia over its atherosclerotic core.

### 4.1 Limitation of the study

The limitations of this study lie in the retrospective analysis of prospectively collected data, which has a lower impact than randomized comparison. The potentially protective effects of any long‐term statin use prior to surgery remain to be seen because the time of any statin administration before CEA was not documented prospectively and therefore could not be included in this analysis. It has been recognized that statin treatments lasting more than 5 days reach a plateau in terms of their vascular pleiotropic effects (Laufs et al., 2001), though some authors have documented that a 3‐year treatment period is needed in order to gain any benefit in terms of stroke reduction (Heart Protection Study Collaborative Group, 2004). All statins were considered as one, irrespective of their dosage, and it was assumed that they have an equal effect on outcome, so we could not establish whether different statins and/or different dosages were more effective than others. In addition, due the nature of this analysis, daily patient compliance to prescribed therapy and changes during follow‐up time including new introduction and duration of statin drugs were not accurately recorded. It is noteworthy that, although the size of our sample of patients was by no means small, the low overall incidence of perioperative adverse events probably prevented this study from having the power needed for any differences to reach statistical significance. Finally, although there is a common conviction that analyzing data from a single institution may be of limited value because they represent the experience of investigators preparing the report (Rothwell & Warlow, 1995), our findings also reflect the experience of a single surgeon: while this has advantages in terms of consistency in the surgical/anesthetic technique, it may pose problems of reproducibility.

## 5 Conclusions

The results of this observational study show that using statins prior to CEA did not affect the risk of perioperative ischemic events and death, most likely due to the extremely low overall incidence of perioperative complications. Since the beneficial effects of statins in the perioperative period remain uncertain, and there is insufficient information to support the initiation of statin treatment as a risk‐reduction strategy in many vascular procedures, including CEA (Antoniou et al., 2015), large‐scale studies are needed to fully establish patient's optimal medical management prior to surgery.

## Conflict of Interest

None declared.
